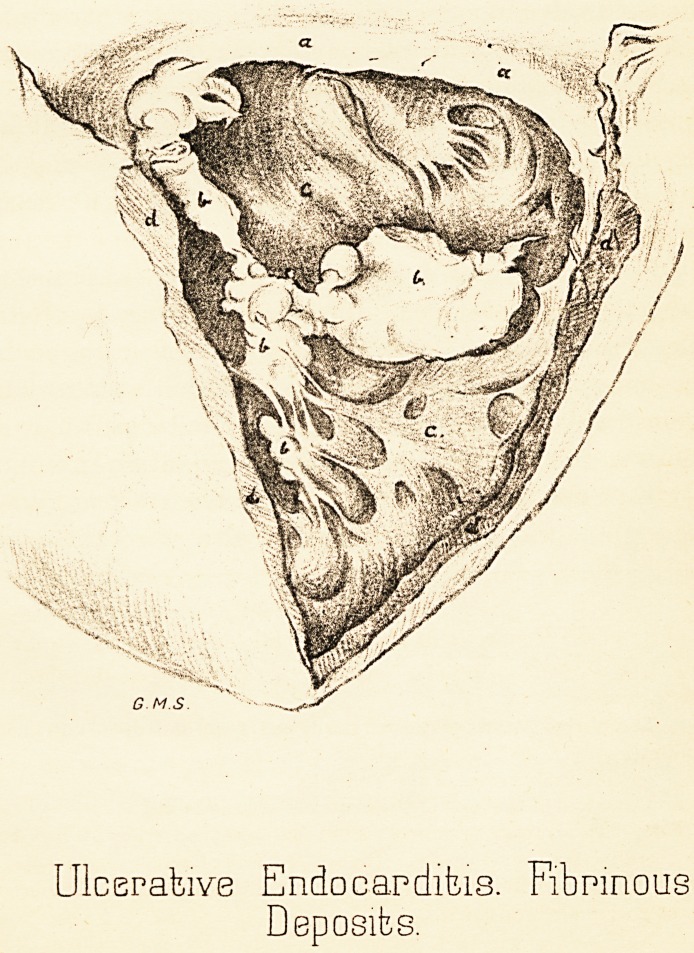# Ulcerative Endocarditis of the Tricuspid Valve. Extensive Fibrinous Deposits

**Published:** 1883-07

**Authors:** J. E. Shaw


### ULCERATIVE ENDOCARDITIS OF THE TRICUSPID VALVE. Extensive Fibrinous Deposits. By J. E. Shaw, M.B.

E. P., æt. 23, single woman, worker in a stay factory, was admitted to the Bristol Infirmary, January 10th, 1883, complaining of rheumatic pains and weakness.

*Previous History.*—Remembered having no previous illnesses. Five months ago, after having got wet several times in going to work and then sitting in her damp clothes, she suffered from pains and swelling in her hands, elbows and shoulders, and was hardly able to walk to her work. Seeking advice she was told that she had rheumatism, was given some medicine, and ordered to stay in bed, but getting no better, in the course of a few days she got up and went to work again, after which the complaint gradually got better. A few weeks ago she was in bed again with her ankles and knees swollen and painful, but at the end of a week she was able to get up and go out, and remained fairly well until four days before admission, when she was taken ill again with pain in her shoulders, back and stomach, lost her appetite and had shivering fits; she then took to bed again and remained there until admission to the Infirmary.

*Family History.*—Her mother was "consumptive" and had dropsy; several of her brothers and sisters were said to have died of consumption.

*State on admission.*—She presented the appearance of a fairly nourished but very anæmic young woman; skin sallow and rather moist, no swelling in any of the joints. *Temperature*, 99°; *pulse*, 136; *respirations*, 30, with obvious subjective dyspnœa. No abnormal physical signs to be found in connection with the *lungs*. The *heart's* impulse was seen to be diffused over a considerable area on a horizontal line with, and internal to the nipple, the apex-beat being very slightly marked. On auscultation there was a bruit loudest over the body of the heart, heard also at the apex, apparently systolic in time, of a blowing character, and the first sound seemed to be reduplicated; there was no independent murmur generated at the base. Very marked pulsation was visible in the carotid arteries, but there was no pulsation present in the jugular veins. *Tongue* large, pale and moist.

The progress of the case during the next month presented no fresh feature of importance. She suffered much from nausea, vomiting and flatulence, especially after mixtures containing digitalis and aconite, which were given with the purpose of reducing the rapidity of the heart's action. She had a troublesome dry cough, without expectoration, and considerable shortness of breath even without any exertion. Her temperature ran the course of an irregular intermittent, and rigors occurred on the 11th and 13th of January, and on the 9th and 12th of February.

February 15th.—Some œdema of feet and ankles appeared to-day for the first time.

February 17th.—A rigor between 4 p.m. and 8 p.m.

February 18th.—Some slight harshness of breathing detected below the right clavicle, and occasional sibilus is to be heard over the lung lower down in front. Her cough is very loud, barking and frequent, attended only by an exceedingly scanty expectoration of clear frothy mucus, with which there has never been any hæmoptysis. Has been complaining of rheumatic pains in her shoulders again this week. Œdema of feet and ankles slightly increased, and there is now a small quantity of fluid in the peritoneal cavity. The liver is symmetrically enlarged, prominent, smooth, tender to palpation; the lower border is about an inch and a half above the level of the umbilicus, while the upper border reaches to the level of the xiphoid cartilage. In the epigastrium there is very strong pulsation, which is communicated to the liver. A well marked systolic murmur is heard loudest at the xiphoid cartilage, though it is diffused over a considerable area, both to the right and left of the mid-sternal line, but more to the latter than to the former; a systolic murmur is also faintly audible at the inferior angle of the left scapula. No venous regurgitation or distension above the valves in the external jugular veins at the root of the neck; there is considerable dyspnœa with tendency to faintness, and pain on coughing in the left side and chest is much complained of. The various remedies—codeia, hydrobromic acid, henbane, &c., which had been employed to allay the violence of the cough had quite failed to do so, but had increased the tendency to vomit. She was therefore placed upon a simple mixture of hydrocyanic acid and cardamoms, with a chloral draught at night when necessary. *Pulse*, 120.

February 21st.—Œdema of feet and legs rather increasing; *urine* contains no albumen but a great excess of uric acid. *Pulse*, 128.

February 22nd.—*Urine*, sp. gr. 1015, no albumen; sleeps better since she had the sleeping draught. Œdema of feet and ankles rather less. Has no pain in chest, but cough is extremely troublesome. Is decidedly weaker in herself. *Pulse*, 122.

February 26th.—A good deal of fluid in peritoneum; liver is not quite so tender to touch and reaches to about two inches above umbilical level. On auscultating the heart a cantering rythm is heard as if composed of a reduplicated first sound and one second sound; there is no murmur generated at the valves at the base but a loud murmur is still audible over the right ventricle, and the apparent reduplication of the first sound was probably a short presystolic murmur which could not be distinctly recognised as such on account of the rapidity of the heart's action. Considerable œdema of feet and ankles. She had rigors again last night, *without* elevation of temperature, followed by sweating. *Urine*, sp. gr. 1015; smoky, a trace of blood present, and albumen for the first time one-twelfth. The occurrence of blood in the urine, without *material* diminution in the quantity excreted, I presumed to be due to an embolic infarct having occurred in one kidney. I was therefore led to change the opinion I had hitherto held, that the endocarditic process was situated in the right chambers of the heart, for the view that it affected the left chambers and valves, and that the pulmonary incompetence, though so emphatically marked by murmur, was simply due to the ordinary mechanical dilatation secondary to lesions of obstruction and incompetence in the mitral valve. *Pulse*, 120.

February 27th.—At 3 p.m. yesterday, when sitting up in bed talking to a friend, she began coughing very violently, soon after which she vomited and felt great pain and fluttering at the heart, causing acute suffering, dyspnœa and cyanosis; she became insensible, and remained so for half an hour; nitrite of amyl, which was administered, did not materially relieve the anginous pain. Complains to-day of feeling very weak. *Pulse*, 100; feeble, irregular and very compressible. *Urine* acid, sp. gr. 1015, blood present, albumen one-eighth, no casts.

February 28th.—Feels somewhat better, but has had yesterday and to-day a sensation of "fluttering at the heart" several times, with attacks of anginous pain also. Did not sleep well last night. A well marked thrill is to be felt over the right ventricle, apparently presystolic in point of time, but owing to the rapidity and irregularity of the heart's action it was impossible to determine with precision. *Pulse*, 100; weak, compressible and irregular. *Tongue* large, moist and clean. *Urine* acid, sp. gr. 1016; rather more blood present, albumen one-sixth, no casts. Digitalis was tried again in ♏v. doses of tincture combined with chloroform and ammonia, and belladonna ointment was applied to the left side to relieve the pain felt there in coughing.

March 1st.—The mixture containing digitalis ordered yesterday induced vomiting. As the urine was plentiful in quantity and the absence of casts from the secretion led me to believe that the hæmaturia was not of an acute inflammatory nature pills containing opium and digitalis, gr. $\frac{1}{2}$ each, were ordered every four hours, as the heart's action was weak and rapid, and she was suffering from great uneasiness. The intellectual faculties were unimpaired. *Pulse*, 108.

March 2nd.—Except for a slight diminution in quantity of urine and slight increase of albumen, she remained in much the same condition. *Pulse*, 100.

March 3rd.—No urine having been passed this morning the pills were stopped, and pulv. jalapæ co. 30 grs., ordered. At time of visit she was quite sensible but said she felt very sleepy, and unless roused, paid no attention to what was going on around her. The rapidity of the heart's action having considerably diminished we could observe with distinctness the perfect presystolic thrill over the right ventricle; the murmurs had almost disappeared, but the cantering rythm of the heart-sounds was very pronounced, produced, as it seemed, by a double first sound and a single second sound. *Pulse*, 80.

In the evening she died from failure of the heart's action, the temperature before death falling to 96°.

*Autopsy* performed twenty hours after death. Much œdema of lower extremities and vulva. *Thorax* only allowed to be opened. A pint or more of fluid in the left pleura. Both lungs decidedly emphysematous, especially at the upper borders and apices; much œdema also of the vesicular structure. In the left lung there were four embolic infarcts, two at the base, one near the root and one at the apex; none of these seemed very recent, being partially decolorised, firm, and presenting no appearances of suppuration in themselves, nor was there inflammation of the tissues around them. In the right lung there was one recent infarct situated on the anterior edge.

The *pericardium* contained about half a pint of clear fluid; there was no lymph or other sign of pericarditis.

The *heart* weighed 14 oz., the right ventricle was much distended. On laying open the right auricle and removing the coloured clot it contained there was seen protruding from below through the tricuspid orifice a rounded white body projecting into the cavity of the auricle, about the size of a small walnut, smooth on the surface but somewhat lobulated. On laying open the right ventricle from its posterior aspect (Pl. VI.) and clearing away the contained clot, this body was seen to be springing from a chorda tendinea uniting the posterior musculus papillaris to the anterior flap of the tricuspid valve, upon which, as well as upon other chordæ tendineæ, were numerous similar firm white "vegetations" resembling in shape and size white currants. The septal flap of the valve was mostly destroyed by the ulcerative process, only ragged strips of it being left attached to its insertion. The inferior flap was unaffected by the process. From the endocardial lining of the front of the ventricle itself in one place there was a small "vegetation" developed. The ventricle was completely filled with "passive" clot, dark-red and soft in the centre, becoming paler and firmer towards the endocardium, against which it was colourless, tough and fibrillated. It however stripped off quite cleanly and easily from the "active" clot of the "vegetations." The endocardium of the right auricle was healthy, as also were the endo-

---

EXPLANATION OF DRAWING.

*The right Ventricle of the Heart laid open from behind :*

*a.* The auriculo-ventricular orifice,—"a" placed upon the shrunken anterior flap.

*b.* The fibrinous "vegetations" developed from and upon one chorda tendinea.

*c.* The internal surface of the ventricle.

*d.* The edges of the wall of the ventricle where divided.

N.B.—The drawing was made from the specimen when it had been preserved in spirit some weeks. The relative size of the "vegetations" is therefore considerably diminished, due to the shrinking they had undergone from the action of the spirit.

Plate VI.

Ulcerative Endocarditis. Fibrinous Deposits.

cardium of the left auricle and ventricle, and the pulmonary, mitral and aortic valves.

The kidneys, liver and spleen were removed by rupturing the diaphragm. The *liver* presented a typically "nutmeg" condition. Into the substance of the cortex of the *kidneys* were numerous punctiform hemorrhages—the source of the blood in the urine during the latter days of life. *Spleen* was enlarged to nearly twice the normal size and was firm in texture. In other respects these viscera were normal. No infarcts existed in any organ besides the lungs. The brain and remaining viscera were not examined. There was no purpuric or other eruption observed anywhere during life or after death except the before mentioned hemorrhages into the cortex of the kidneys.

*Remarks.*—This case is of interest as being a very good example of ulcerative endocarditis of the septicæmic type, and possesses a special interest as an example of acute endocarditis affecting one of the right chambers of the heart. This, when it occurs in adult life, is much more frequently of the "ulcerative" variety than of the "verrucose"; and "vegetations" springing from parts of the endocardium other than that covering the valve-flaps, such as existed here, seem to be found in the ulcerative variety of endocarditis only.

We had here the most frequent causal (or concomitant) malady—rheumatism. The lesions in the tricuspid valve were all recent, so that in this instance the acute ulcerative process was not engrafted upon a chronic endocarditis, or its consequences, as is most usually the case.

## Figures and Tables

**Figure f1:**